# Quantum Phase Stability in Human Cognition

**DOI:** 10.3389/fpsyg.2019.00929

**Published:** 2019-04-30

**Authors:** Ilya A. Surov, Sergey V. Pilkevich, Alexander P. Alodjants, Sergey V. Khmelevsky

**Affiliations:** ^1^Laboratory of Quantum Cognition and Intelligence Systems, ITMO University, Saint Petersburg, Russia; ^2^Mozhaisky MSA, Saint Petersburg, Russia

**Keywords:** quantum cognition, quantum phase, decision making, irrational behavior, predictive modeling

## Abstract

Quantum approach to human cognition and behavior suffers from a so-called phase problem—lack of means to determine the phase parameter of quantum models before the experiment, which deprives them of predictive power and most of their potential practical impact. We report an empirically supported hypothesis which can help to resolve the issue. According to the hypothesis, the quantum phase between unresolved cognitive alternatives in a family of similar decision making situations is nearly constant across national, linguistic, and cultural backgrounds of subjects. If confirmed, the quantum phase stability phenomenon supplements the quantum model of decision making endowing it with predictive power. This possibility is demonstrated in the testing experiment where irrational behavior within previously unexplored social group could be probabilistically predicted with high accuracy.

## 1. Introduction

The probabilistic and irrational nature of human decision making was recognized as object of scientific study in the 1970s when phenomena like question order effect in surveys and ubiquity of various behavioral heuristics were identified (Tversky and Kahneman, 1973, 1974; Sudman and Bradburn, 1974; Kahneman, 2011). A renowned answer to this challenge—the prospect theory—allowed to model several typical inconsistencies of human choice by evaluating subjective attitudes toward possible gains and losses instead of the absolute monetary utilities (Kahneman and Tversky, 1979; Oliver, 2018). Subsequent upgrades of the prospect theory elaborated on subjective nature of behavioral utility, accounting for simple heuristics, mental sampling, and other cognitive human traits (Erev et al., 2017). Although locally efficient, all these models are of heuristic nature, capturing particular decision making algorithms in *ad hoc* mathematical constructions.

A conceptual way to model probabilistic and irrational behavioral phenomena adopts to this end quantum theory, initially developed to describe probabilistic processes in atom-scale physics (Orlov, 1981; Peres, 2002; Aerts, 2009; Busemeyer and Wang, 2015; Khrennikov, 2015). This approach builds on a self-consistent methodological ground of quantum theory and benefits from its built-in contextual probabilistic nature, allowing production of quantitative models for a variety of behavioral phenomena which compromise the classical rational-agent paradigm: the question order effects, risk and ambiguity aversions, altruistic cooperation, social and market instabilities, conjunction-, disjunction- and other “fallacies” of human logic (Khrennikov, 2010; Busemeyer and Bruza, 2012; Haven and Khrennikov, 2013, 2017; Aerts et al., 2016b). This seemingly unexpected efficiency of quantum approach to human cognition and behavior supports a conjecture that the mathematical formalism of the quantum theory constitutes the basic calculus of contextual probabilistic phenomena in nature (Gabora and Aerts, 2005).

### 1.1. The Quantum Approach

In quantum framework, a decision making process is described as a transition of a subject from an indefinite to definite cognitive state in relation to the considered set of decision alternatives. The latter, if mutually exclusive, form an orthogonal basis in the complex vector space where the cognitive state Φ of the subject is represented as vector (or a subspace of larger dimension) |Ψ〉. In that state, the probability of producing a particular decision *A*, associated with the corresponding cognitive state |*A*〉 is given by squared modulus of the complex-valued overlap 〈Ψ|*A*〉, known in quantum theory as *amplitude of transition* between the corresponding states. Description of human thinking in terms of transition amplitudes allows indistinguishable cognitive alternatives to interfere, producing deviations from classical set-theoretic probability calculus corresponding to the rational boolean logic.

Being complex numbers, transition amplitudes are characterized by two real values, the amplitude and the phase. Contrary to the amplitude, the phase of the transition is not determined by directly measurable probability of the corresponding decision. This is a distinctive feature of the quantum theory where the phase does not have a well-defined observable (Lynch, 1995). It is this elusive nature of the quantum phase, constituting the main hurdle in development of the quantum approach to human cognition and behavior, which is the target of the present work.

A simple behavioral experiment where cognitive interference manifests itself is known as a *two-stage gambling task* (Tversky and Shafir, 1992). It is a decision to play or not to play in a fair game of dice given that outcome of the previous round is won, lost, or unknown with 50/50 chance; amounts of possible gain and loss are 200 and 100$, respectively. Measured quantities are statistical probabilities of the positive decision (A) in all three cases, henceforth denoted by *p*(*A*|*B*_1_), *p*(*A*|*B*_2_), and *p*(*A*) where *B*_1_ and *B*_2_ indicate “won” and “lost” conditions.

In quantum approach, conditions *B*_*i*_ are represented by vectors |*B*_*i*_〉 in the same vector space as decision |*A*〉 and cognitive state |Ψ〉. Because conditions *B*_*i*_ are mutually exclusive and $\underset{i}{\sum }p\left(B_{i}\right)=1$, the amplitude of reaching the decision *A* from cognitive state Ψ expands as

(1) $$\begin{array}{r} \langle \text{Ψ}|A\rangle =\underset{i}{\sum }\langle \text{Ψ}|B_{i}\rangle \langle B_{i}|A\rangle . \end{array}$$

**Figure 1:**
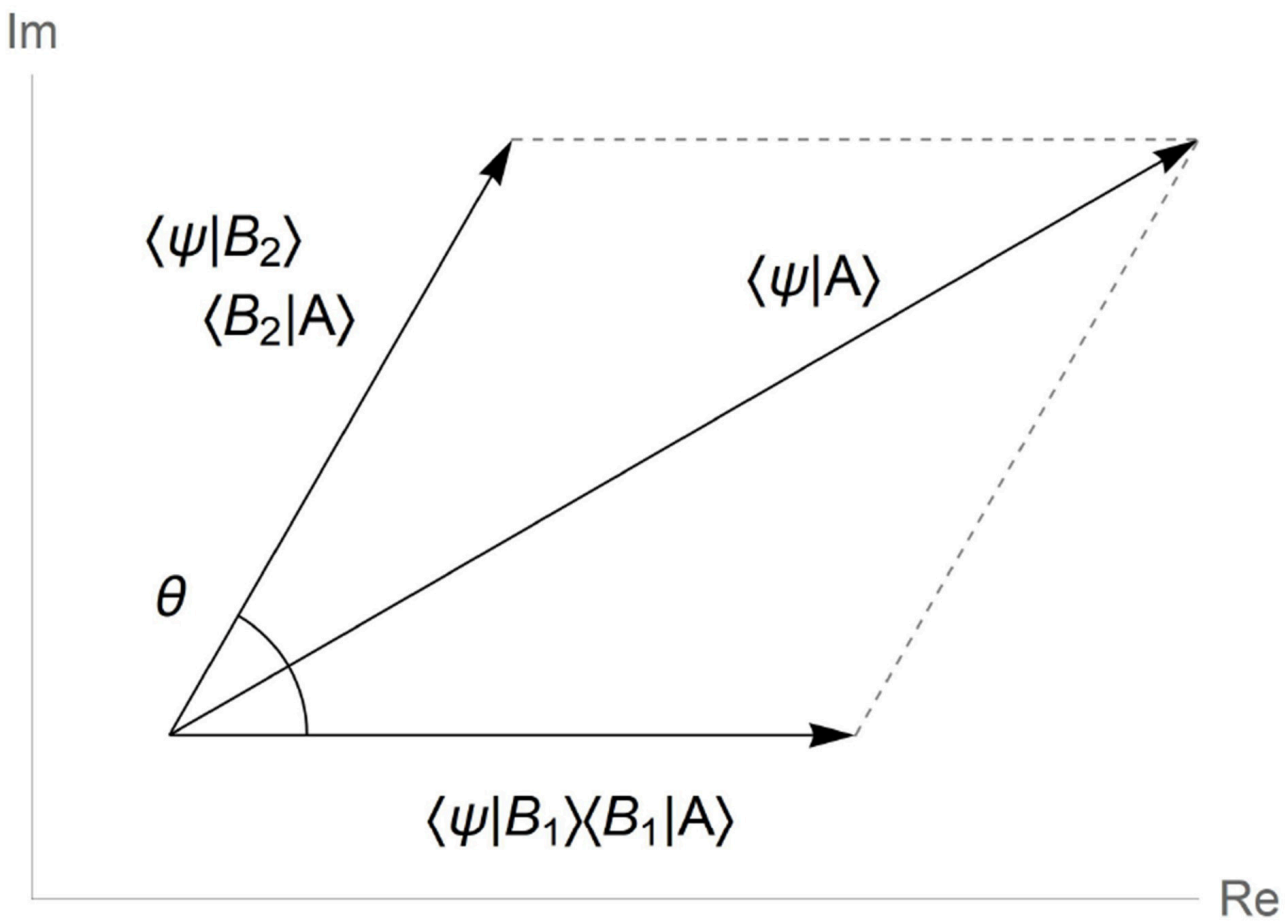
Quantum law of total amplitudes visualized in vector form. Phase θ is the angle between the interfering amplitudes of unresolved cognitive alternatives 〈Ψ|*B*_*i*_〉〈*B*_*i*_|*A*〉 in complex plane.

Formula (1), sometimes called as *law of total amplitude*, can be visualized as shown in Figure 1, where complex-valued amplitudes 〈Ψ|*B*_*i*_〉〈*B*_*i*_|*A*〉 are represented as vectors on the complex plane. The difference between phases of the two complex amplitudes is then mapped to the angle θ between the two vector summands.

Statistical probability of taking decision *A* is

(2) $$\begin{array}{l} p_{\text{quant}}(A)={\left|\langle \Psi |A\rangle \right|}^{2}=\sum_{i}p(A|B_{i})p(B_{i})+2\delta \cos\theta ,\\ \;\delta =\sqrt{p(A|B_{1})p(B_{1})p(A|B_{2})p(B_{2})},\\ \;\theta =\text{Arg}\left[\langle \Psi |B_{1}\rangle \langle B_{1}|A\rangle {\langle \Psi |B_{2}\rangle }^{*}{\langle B_{2}|A\rangle }^{*}\right]. \end{array}$$

where * is complex conjugation sign. The first summand in (2) reproduces the classical law of total probability, while the second one is known as interference term between the two transition amplitudes characterized by magnitude δ and *interference phase* θ. Equation (2) establishes a connection between experimental probabilities *p*(*A*|*B*_1_), *p*(*A*|*B*_2_), and *p*(*A*), characterized by interference phase θ.

Solving (2) for θ allows one to fit the measured values *p*(*A*|*B*_1_), *p*(*A*|*B*_2_), and *p*(*A*) into the mathematical structure just sketched. In common practice, this fitting step finalizes the building of the quantum model for a particular experiment (Khrennikov, 2010; Busemeyer and Bruza, 2012).

The interference term in (2), constituting the difference between the quantum and the classical probability models, is necessary to fit all existing experimental results on the two-stage gambling task. This indicates that logic of decision making in the corresponding ensembles of subjects is not classical, but “quantum-like” (Khrennikov, 2015).

### 1.2. The Quantum Phase Problem

Though the development of the above quantum model is a great step forward in modeling of human behavior, its application in the present form is mostly limited to conceptual explanations of the considered phenomena accompanied by a posteriori fitting of the experimental data. The reason is that currently there is no way to find the interference phase θ before the experiment; consequent treating of θ as free fitting parameter deprives the above model of predictive power and thus of most of its potential practical value. At present, this situation is typical for most of the current quantum models in cognition and decision making (Khrennikov, 2010; Busemeyer and Bruza, 2012); (Haven and Khrennikov, 2013).

Below we report a hypothesis which addresses this quantum phase problem. The hypothesis is based on the observation that the value of quantum phase between interfering alternatives in the two-stage gambling experiments conducted in diverse national, linguistic, and cultural environments remains approximately constant. If confirmed, this new regularity supplements the standard quantum model of decision making allowing one to use it in a predictive manner. Further, we report results of the testing experiment supporting the proposed hypothesis.

## 2. The Hypothesis

### 2.1. Analysis of the Existing Data

The hypothesis becomes evident upon the analysis of the existing two-stage gambling experiments summarized in Table 1 (Tversky and Shafir, 1992; Kühberger et al., 2001; Lambdin and Burdsal, 2007). Notation of conditional and prior probabilities to play *p*(*A*|*B*_*i*_) and *p*(*A*) is as above.

**Table 1:** Existing experimental data on the two-stage gambling task.

**No**.	**Description**	**Won** ***p(A|B_1_)***	**Lost** ***p(A|B_2_)***	**Unknown** ***p(A)***	**θ**
1	One subject single decision	0.69	0.57	0.38	114°
2	All decisions with a week break	0.69	0.59	0.35	117°
3	All decisions considered at once	0.71	0.56	0.84	71°
4	Rep. of 1	0.60	0.47	0.47	97°
5	Rep. of 1 with 10 times smaller bets	0.83	0.70	0.62	101°
6	Rep. of 3 with 10 times smaller bets	0.80	0.37	0.43	107°
7	Rep. of 1 with 10 times smaller real bets	0.68	0.32	0.38	105°
8	Rep. of 1	0.64	0.47	0.38	109°
9	Rep. of 1, weighted dice, different bets	0.53	0.38	0.24	119°
10	Rep. of 1, weighted dice, different bets	0.73	0.49	0.60	91°
11	Setup of exp. 8	0.30	0.24	0.17	112°

*Columns 3-5 show measured probabilities to play the game, given that the previous round is won, lost or unknown with 50/50 chance. Phase θ fitted for each experiment according to (3) is shown in the rightmost column. Experiments 1–3: Tversky and Shafir (1992). Experiments 4–7: Kühberger et al. (2001). Experiments 8–10: Lambdin and Burdsal (2007). 11: our experiment*.

The difference between *p*(*A*) and average of *p*(*A*|*B*_1_) and *p*(*A*|*B*_2_), which quantifies violation of the classical law of total probability, in quantum model is accounted by the second summand in Equation (2). In line with the common practice we use this relation rewritten as

(3) $$\begin{array}{r} \cos\theta =\frac{p\left(A\right)-\sum_{i}p\left(A|B_{i}\right)p\left(B_{i}\right)}{2\delta } \end{array}$$

to fit the values of θ for all known to us two-stage gambling experiments listed in Table 1.

**Figure 2:**
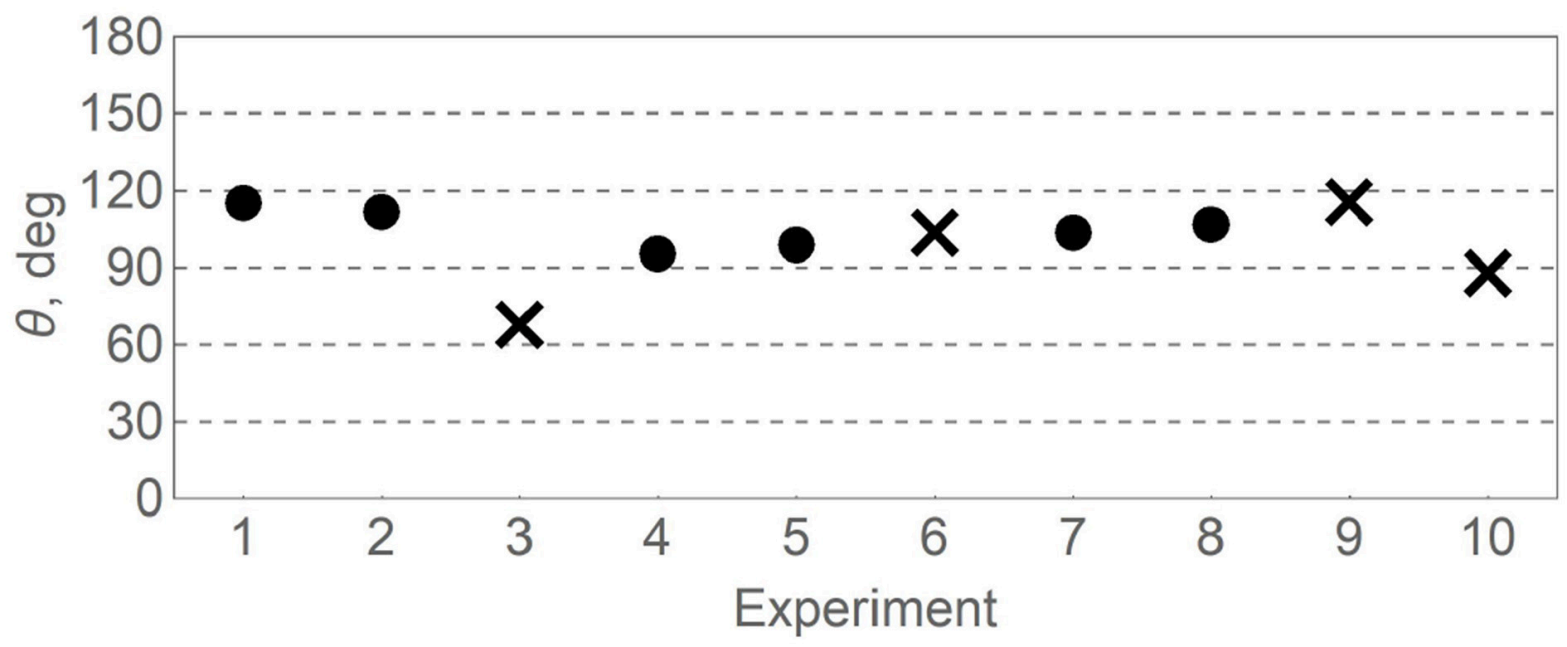
Quantum phase θ fitted for the existing two-stage gambling experiments (Table 1) from the quantum model (3). Crosses indicate cases of strongly modified setup (3 and 6) and different task specification (9 and 10).

Results of the calculation are shown in the Figure 2. Clearly, the distribution of θ in the interval [0°, 180°] is far from uniform as expected if θ would be a meaningless fitting parameter. Instead, the distribution is sharply peaked around θ ≈ 100°. For all but one experiment θ is larger than 90° which corresponds to moderately negative interference responsible for decrease of *p*(*A*) from rational expectation given by (2) with θ = 90°.

The only case deviating from this trend is experiment 3 in which all three decision tasks were presented on the same instruction page, presumably encouraging subjects for rationally-consistent decision making (Tversky and Shafir, 1992). This setup strongly differs from others where decisions *A*, *A*|*B*_1_ and *A*|*B*_2_ were separated in time or assigned to different subjects. Because of that we exclude experiment 3 and its repetition 6 from the following analysis. We also drop experiments 9 and 10 which used weighted dice with different win/lose probabilities and different payoff ratios (Lambdin and Burdsal, 2007).

The remaining six experiments all share the same probability and payoff ratios (50% chance to lose some amount, otherwise win twice as much), although absolute payoff amounts, as well as their real or imaginary realization, vary. Averaging over these experiments (dots in the Figure 2) gives estimations of the interference phase

(4) $$\begin{array}{r} \theta_{\text{est}}=107\pm 7{}^{\circ}, \end{array}$$

where 7° is one standard deviation from the mean.

### 2.2. The Idea

We conjecture that such compactness of the phase distribution is not a coincidence but a regularity; in fact, probability of six random numbers from uniform distribution on the interval [0°, 180°] to fall into a window of 20° is <1/500,000.

Our hypothesis constituting the core of the present work is that the quantum interference phase θ in quantum model of the two-stage gambling task is a constant largely insensitive of cultural, national, and linguistic identities of subject group.

An experimental test of this hypothesis is presented below.

## 3. Testing Experiment

Direct way to verify our hypothesis which we adopt here is to conduct the same behavioral experiment in a social group with nation, country, language and culture different from these in previous experiments:

1. Tversky and Shafir (1992): Stanford University, US;
2. Kühberger et al. (2001): Salzburg University, Austria;
3. Lambdin and Burdsal (2007): Wichita State University, US.

Our subjects (students of a Saint-Petersburg technical high school, Russia) differ from subjects of these experiments in all four parameters. Setup of the experiment is close to that of experiment 8 in Table 1 (Lambdin and Burdsal, 2007), see section Materials and Methods for details.

In the conditions of definite “won” and “lost” result of the previous round, the statistical probabilities to play the game are found to be *p*(*A*|*B*_1_) = 0.30 and *p*(*A*|*B*_2_) = 0.24, respectively.

According to the hypothesis, when the outcome of the previous round is unknown, the quantum phase between unresolved and interfering cognitive alternatives *B*_*i*_ is expected to lie in the interval (4). Equation (2) then *predicts* proportion of the subjects who decide to play in the “unknown” condition as

(5) $$\begin{array}{l} p{\left(A\right)}_{\text{predict}}=\frac{p(A|B_{1})+p(A|B_{2})}{2}+\\ \;+\sqrt{p(A|B_{1})p(A|B_{2})}*\cos\theta_{est}=0.19\pm 0.03. \end{array}$$

Interval (5) can be compared with the range 0.0016 < *p*(*A*) < 0.54 which is expected from (2) if without the stability hypothesis phase θ can take any value from 0° to 180°.

**Figure 3:**
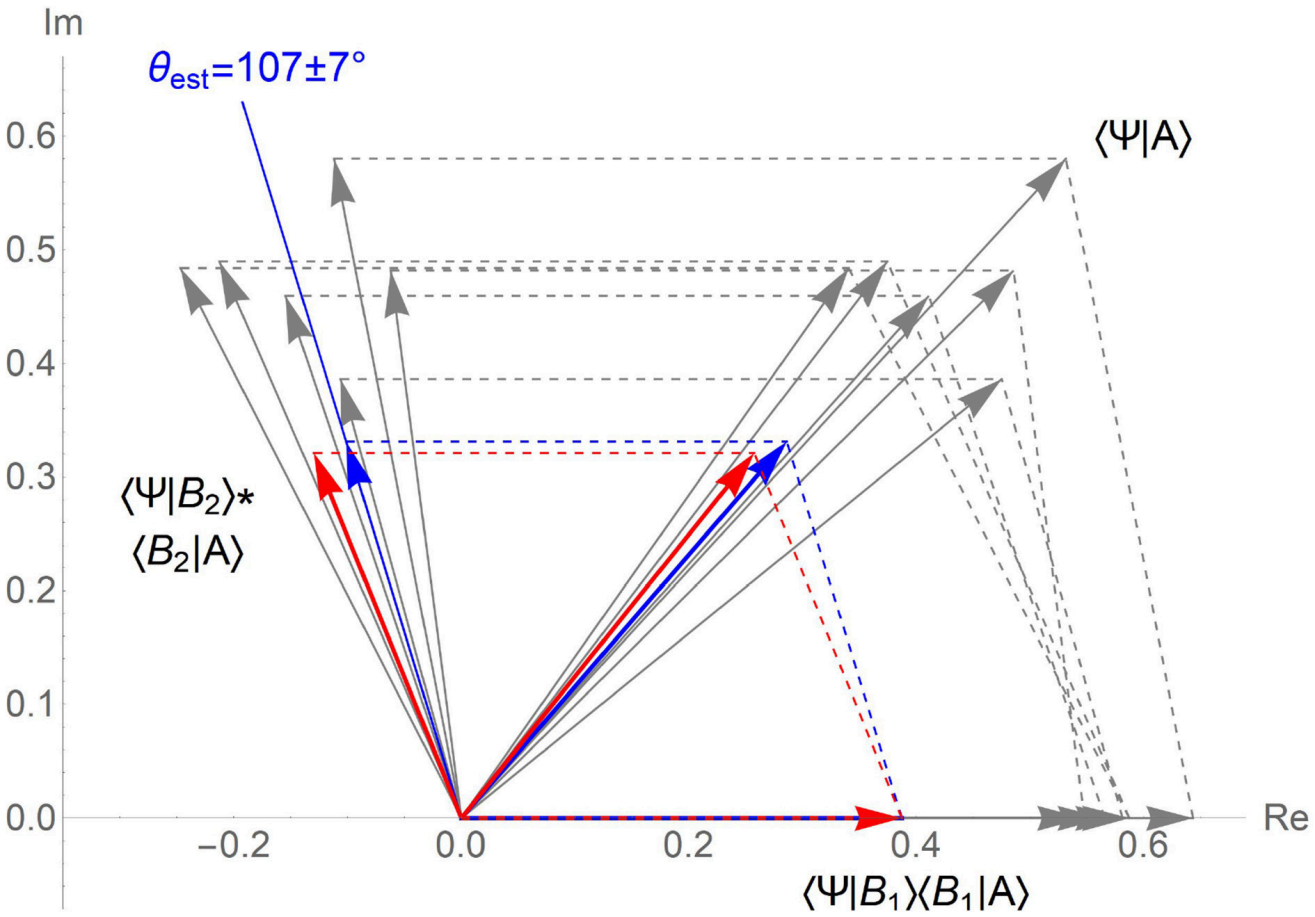
Verification of the quantum phase stability hypothesis and quantum scheme for predictive behavioral modeling. Quantum models of the relevant existing experiments and of the testing experiment in vector amplitude form (Figure 1) are shown in gray and red. Proximity of directions 〈Ψ|*B*_2_〉〈*B*_2_|*A*〉 and 〈Ψ|*A*〉 for different experiments indicates agreement with the hypothesis. Application of the quantum phase stability to prediction of our experimental result is shown in blue. Vector amplitudes 〈Ψ|*B*_*i*_〉〈*B*_*i*_|*A*〉 are computed from the measured probabilities and the expected phase value $\theta_{\text{est}}=107\pm 7{}^{\circ}$. Squared length of the sum of these vectors 0.19 ± 0.03 gives correct prediction of the target experimental probability *p*(*A*) = 0.17.

The experimentally measured value *p*(*A*) = 0.17 agrees with prediction (5). Phase parameter fitted to our data according to (3) $\theta_{\text{exp}}=112\pm 8^{∙}$ also falls into the expected confidence interval (4). This result supports the phase stability hypothesis as visualized in the Figure 3.

## 4. Discussion

### 4.1. Culture-Dependent Behavior

As the expected profit of the game (200−100*$*)/2 = 50*$* is positive and independent on the outcome of the previous rounds, rationally thinking subjects would doubtlessly play in all three conditions producing statistical probabilities *p*(*A*) = *p*(*A*|*B*_1_) = *p*(*A*|*B*_2_) = 1. The fact that these probabilities measured in our experiment are 2–3 times smaller than corresponding values recorded previously then means that our subjects are more active users of irrational behavioral heuristic known as *risk aversion* than subjects of previous experiments (Hillson, 2007; Oliver, 2018).

Mechanisms responsible for such distinction of attitudes toward games of chance may have profound cultural and eventually environmental origins (Bechtel and Churman, 2002; Meyer et al., 2009) which shaped the regional psychotypes in the US, Western Europe, and the North-West of Russia. For example, higher price of unfortunate outcome of chancy behavior in less forgiving northern climates evolutionally favors stronger risk aversion observed in our subject group.

### 4.2. Predictive Behavioral Modeling

A traditional approach to behavioral prediction in the two-stage gambling task would aim to address decision probabilities *p*(*A*|*B*_1_), *p*(*A*|*B*_2_), and *p*(*A*) individually. Variability of data in Table 1 indicates that these values are sensitive to hardly formalizable features of particular decision situation. Moreover, our experiment shows that individual decision probabilities are also dependent on cultural identity of subjects (see also section 4.1). As these factors contain uncountable degrees of freedom, their direct prognosis is practically impossible.

Quantum phase stability hypothesis suggests another approach to predictive behavioral modeling. It consists in supplementing the standard quantum decision model (section 1.1) with the phase stability relation established beforehand. Our experiment shows that, this approach potentially allows for probabilistic prediction of human irrational decision with relative error of ~0.1 (Figure 3). If attained for other quantum models in human cognition and behavior, this level of prediction fidelity would constitute a breakthrough in the present day psychology and social science.

The approach to behavioral modeling we envision is fundamentally different from the traditional one discussed above. It utilizes experimental input from a set of related behavioral situations integrated into the quantum theoretical framework which encapsulates both rational and irrational regularities of human cognition. Results of our work imply that such regularities may not only address independent decision probabilities, but can also encompass set of them as a whole (see section 4.3). Our experiment shows that regularities of this latter type can be more robust to both external and internal decision factors. Thank to this stability, these “hidden” regularities can be transported to previously unexplored decision situations and thus are potentially more useful in predictive behavioral modeling.

### 4.3. Behavioral Semantics

Quantum models of both elementary physical processes and behavior of living systems can be seen as models of *semantics* (or, equivalently, *meaning* and *logic*) which generates the observed data (Aerts, 2010, 2014). Understanding the reported quantum phase stability phenomenon as representation of “hidden” regularity behind interconnected decision acts (section 4.2) naturally fits this view.

In accord with quantum approach to concept modeling (Aerts, 2009; Aerts et al., 2016a), in the two-stage gambling task the “unknown” condition combines the unresolved “won” and “lost” alternatives regarding the outcome of the previous round, thereby creating an individual behavioral case represented in the form of quantum superposition. In this superposition (Equation 1, Figure 1), the quantum phase θ, “hidden” from direct measurement, encodes a stable meaning relation between behavioral possibilities in cognition of a subject. In semantic terms, the quantum phase stability hypothesis reads that *behavioral semantics in the two-stage gambling task is largely insensitive of cultural, national and linguistic variables of subjects*.

Another stable combination of behavioral probabilities established by quantum theory follows from a so-called “QQ equality” linking a pair of related question oder effects (Wang and Busemeyer, 2013; Wang et al., 2014). Like the quantum phase stability phenomenon, the QQ equality allows for apriori prediction of behavioral probabilities which is unexpected from the classical viewpoint. On the other hand, QQ equality is derived as a theorem from the first principles of quantum cognitive paradigm and as such is completely parameter-free. One may say that the QQ model expresses a fundamental semantic restriction imposed on the behavior which can be modeled by standard quantum theory.

#### 4.3.1. Retrieval of Culture Maps

Based on the semantic interpretation of quantum phase stability, one may envision an approach for studying semantics of collective cognition termed as the “Quantum World Wide Web” (Aerts et al., 2018). The above ideas imply that structures of this “QWWW” can be experimentally retrieved as patterns the quantum phase stability relations between entities of conceptual space representing human cognition and culture (Aerts et al., 1994; Gabora and Aerts, 2009). If approved, this methodology can be used to characterize the behavioral algorithmics of different social groups based on the quantitative measure—the quantum cognitive phase.

### 4.4. Verification and Scope

Whereas proximity of the quantum phase values shown in the Figures 2, 3 is unlikely to be generated by coincidence, scope of the hypothesized regularity is yet to be understood. For example, all four families of relevant experiments are conducted in European kind of cultures, with subjects in each case being undergraduate students who represent a narrow slice of society. Testing the quantum phase stability hypothesis in subject groups of diverse ages and education levels including native Asian, African, and South-American cultures is vital to further progress in study of human cognition.

## 5. Materials and Methods

Subjects of our experiment were 85 students of a technical high school between 20 and 25 years of age who agreed to participate in the behavioral study. Each subject was given a notebook-like questionnaire, the first two pages of which contained the following information translated from Russian:

Page 1: *You are invited to participate in behavioral study and answer to a few simple questions. Do not hesitate and select the first answer which comes to the mind*.
Page 2: *Imagine that you take part in the game where you can win $200 or loose $100. Chances to win and loose are equal. Get ready to play!*

Each of the next seven pages contained one of the following tasks, in the text referred to as Won, Lost and Unknown conditions:

Task 1: *Congratulations! You have just won the game. Knowing that, would you like to play one more time?*
Task 2: *Unfortunately, you have just lost the game. Knowing that, would you like to play one more time?*
Task 3: *The game is over, but the results are not yet known. Without knowing the result, would you like to play one more time?*

Order of the tasks was randomized among the subjects.

In that way each subject has successively taken seven decisions. Values *p*(*A*|*B*_1_), *p*(*A*|*B*_2_), and *p*(*A*) were calculated as number of positive decisions in tasks 1–3, divided by the number of times each task was addressed.

## Ethics Statement

This study was carried out in accordance with the recommendations of Code of Ethics of the Russian Psychological Society, Institute of Psychology and Social Work with written informed consent from all subjects. All subjects gave written informed consent in accordance with the Declaration of Helsinki. The protocol was approved by the Ethics Committee of the Institute of Psychology and Social Work, Saint Petersburg.

## Author Contributions

IS proposed the hypothesis, analyzed and interpreted experimental results, and prepared the manuscript. SP developed the experimental procedure, conducted the experiment, analyzed experimental results, and contributed to the manuscript. AA and SK organized the research, designed the experiment, and contributed to the manuscript.

### Conflict of Interest Statement

The authors declare that the research was conducted in the absence of any commercial or financial relationships that could be construed as a potential conflict of interest.
